# Delayed onset acute cerebral venous infarct following meningioma excision: a word of caution

**DOI:** 10.11604/pamj.2020.36.371.17586

**Published:** 2020-08-29

**Authors:** Gautam Dutta, Divya Mahajan, Daljit Singh, Hukum Singh, Anita Jagetia, Arvind Kumar Srivastava

**Affiliations:** 1 Department of Neuro-Surgery, Rajendra Institute of Medical Sciences (RIMS), Ranchi, India,; 2 Department of Radiation Oncology, Guru Govind Singh Medical College (GGSMC) and Hospital, Faridkot, India,; 3 Department of Neuro-Surgery, Govind Ballabh Pant Institute of Postgraduate Medical Education and Research (GIPMER), New Delhi, India

**Keywords:** Meningioma, venous infarct, parasagittal

## To the editors of the Pan African Medical Journal

**Figure 1:**
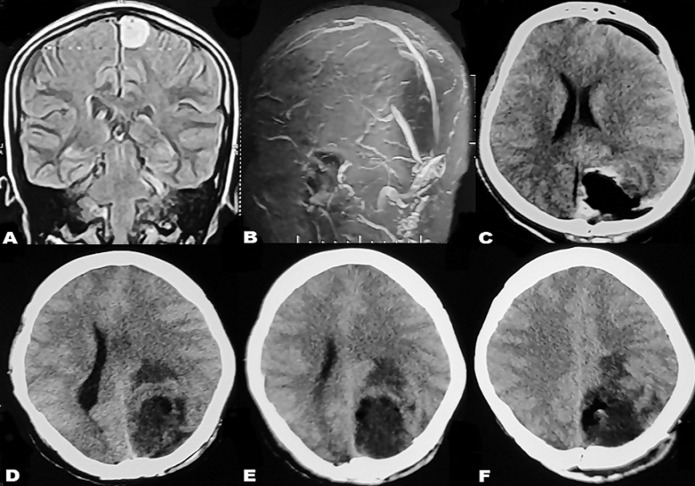
(A) contrast-enhanced MRI brain showing left posterior parasagittal meningioma; (B) grossly normal preoperative MR venogram of the patient; (C) contrast-enhanced CT brain showing gross total tumor excision; (D,E,F) CT brain on 15^th^ post-operative day: extensive cerebral edema with acute parieto-occipital infarct

Clinical symptomatology and prognosis of post-operative cerebral venous infarction (POCVI) are known to be quite variable and little information is available regarding the pathophysiology and management of this potentially catastrophic complication. POCVI usually manifests itself acutely within hours of surgery or it may be chronic. However, delayed onset acute form is not known. We describe one such unusual case. A 45-year-old female patient underwent Simpson grade I excision of her posterior parasagittal meningioma (Figure 1 A and B). No bridging veins were coagulated during the procedure and she tolerated the procedure quite well. There was no evidence of any hypercoagulable state and her coagulation profile was normal postoperatively. Immediate post-operative contrast-enhanced computed tomography (CT) brain revealed gross total tumor excision without any evidence of hematoma (Figure 1 C). Mannitol infusion was given postoperatively for 2 days and her stay in the hospital was uneventful and she was discharged in satisfactory condition on 8^th^ post-operative day. On 15^th^ post-operative day, she was re-admitted with complaints of sudden onset disorientation, nausea and vomiting. On examination, she was drowsy, obtunded, did not follow commands and responded only to sternal rub. Keeping possible differential diagnosis of pulmonary embolism, infarct or delayed surgical site hematoma in mind, complete blood counts, renal and liver function tests and electrolytes were obtained which were all within normal limits. Workup for hypercoagulable state including protein C and S were negative. CT brain was obtained which revealed severe diffuse cerebral edema along with parieto-occipital acute infarct (Figure 1 D, E and F).

Endotracheal intubation was done and mechanical ventilation started along with initiation of anti-edema measures (mannitol and furosemide) and anticoagulants and antithrombotics. A magnetic resonance imaging (MRI) and venography (MRV)/CT venography was planned but could not be performed due to her critically ill condition. She did not respond to the treatment and before decompressive craniectomy could be performed, her condition quickly deteriorated and she died within 24-hrs of admission despite aggressive measures. Cushing *et al*. [1] described parasagittal meningioma as a tumor in which there is no brain tissue between the tumor and superior sagittal sinus. Owing to this characteristic, venous drainage compromise, infarction and neurologic deficit might ensue. Robertson [2] divided venous infarction into two types: the acute form and the chronic form. The acute form manifests in the postoperative period and can be life-threatening. The chronic form manifests itself months or years postoperatively with headache, disequilibrium and visual disturbances due to papilledema. Nakase [3] further divided the acute form into mild and severe types. The mild type has a slow neurological deterioration by gradual thrombus evolution and can be treated conservatively. The patient is usually conscious initially and deteriorates neurologically after a variable lag period ranging from 2 to 5 days. In the severe type, the patient has altered sensorium or focal neurological deficit from the immediate postoperative period and may require aggressive management, which may include decompressive surgery and barbiturate therapy. Our patient developed acute onset infarct on 15^th^ post-operative day which has not previously been described.

## Conclusion

The occurrence of delayed-onset acute POCVI could possibly be related to the hypercoagulable state of the tumor bed leading to thrombosis of the vessels. However, more studies are required to study the pathophysiology and prevention of this apparently virulent condition. In cases of meningioma excision, we urge neurosurgeons to remain vigilant even after an apparently uneventful post-operative course as delayed onset POCVI could be fatal.
